# Supplementary material for: Learning curve and surgical outcome of robotic assisted colorectal surgery with ERAS program
Source: Sci Rep. 2022 Nov 29;12:20566. doi: 10.1038/s41598-022-24665-w (PMC9709162; doi:10.1038/s41598-022-24665-w)
Supplement: Supplementary file 2 — Supplementary Information 2. [file 41598_2022_24665_MOESM2_ESM.zip › RAL Raw data-Ñ[▒K/141/OP_ñw╝╨░O▒Kñσ.pdf]

正式摘要(電子簽章：000000233943907)

請選擇...reporter

臺中榮民總醫院

Operation Note

[redacted]

- 手術日期：2022/01/07
- 手術醫師：蔣鋒帆，Fellow 楊曜榮，CR 陳瑨夫，R 林志安，R 陳威遠，R 陳凱祥
- Anesthesia：GE
- Position：Lithotomy
- Aseptic agents：Soap，Betadine Alcohol，Betadine Aqua

Diagnosis

- Pre-operative diagnosis : Malignant tumor of rectum 8CM
- Post-operative diagnosis : Rectal tumor at middle rectum

Surgical Indication

- tumor resection

Operation

- Robot Assisted Low Anterior Resection + loop ileostomy

Finding

- 1. post polpyectomy scar at middle rectum
  2. Robotic LAR + loop ileostomy was complet
  3. IMA high ligation+
  4. air tie test neg. donut intact
  5. loop ileostomy in LLQ

Procedure

- 1. under GE, the pt was in lithotomy position
  2. the trocar hold was made in periumbilicus open method. the other 5 hole made by punctiure
  3. identify holly plain and dissct. isolated IMA root. ligated with clips
  4. dissect between Gerota fascia and meso rectum. lysis Lt white line
  5. dissect into pelvis area. dissect posterior side of rectum down to pelvis follor levetar ani. anterior side of Denovillor fascia.
  6. resected rectum stump with Stapler 45mm 2 fires.
  7. pull the specimen outside abdomen from midline wd
  8. resect Rectum
  9. embeded envril 22 mm
  10.complete anastomsis with side to end. air tie test neg. donut intact.
  11. loop ileostomy in RLQ
  12.he tolerate whole procedure and extubated.
- Estimated blood loss: minimum
- 器械數量: 正確
- 紗布數量: 正確

Image

- PAINT
  - abdomen

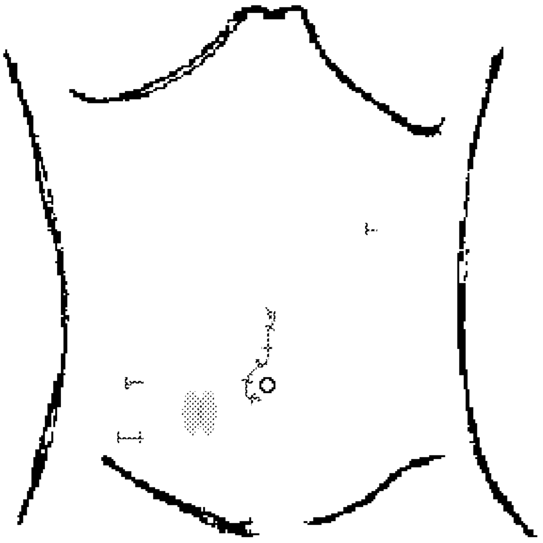

- colon & rectum

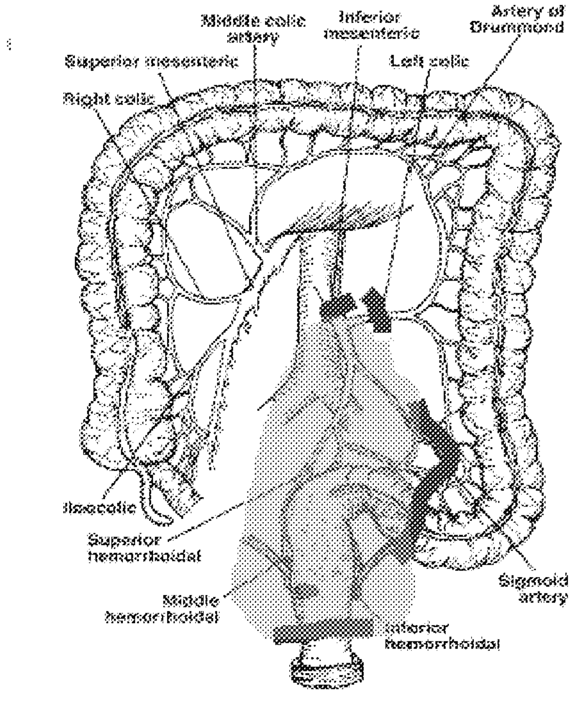

主任 2062F蔣鋒帆 / 醫師 2292G楊曙榮

正式摘要(電子簽章：000000233943907)

請選擇...reporter

doctorRank:B
